# Antibiotics for the Treatment of Patients with Metallo-β-Lactamase (MBL)-Producing Gram-Negative Bacterial Infections

**DOI:** 10.3390/antibiotics14090894

**Published:** 2025-09-04

**Authors:** Matthew E. Falagas, Dimitrios S. Kontogiannis, Dimitrios Ragias, Stylianos A. Kakoullis

**Affiliations:** 1 Alfa Institute of Biomedical Sciences (AIBS), 9 Neapoleos Street, 151 23 Marousi, Athens, Greece; 2 School of Medicine, European University Cyprus, 6 Diogenous Str., 2404 Nicosia, Cyprus; s.kakoullis@euc.ac.cy; 3 Department of Medicine, Tufts University School of Medicine, Boston, MA 02111, USA

**Keywords:** *Acinetobacter baumannii*, aminoglycosides, aztreonam-avibactam, cefiderocol, colistin, *Escherichia coli*, fosfomycin, *Klebsiella pneumoniae*, metallo-β-lactamases, *Pseudomonas aeruginosa*, tigecycline

## Abstract

Infections caused by MBL-producing Gram-negative bacterial pathogens have disseminated worldwide and are associated with considerable morbidity and mortality. We evaluated the published clinical evidence on the use of various antibiotics for the treatment of patients with such infections. The available data suggest the consideration of the new antibiotics cefiderocol and aztreonam-avibactam for the treatment of patients with infections caused by MBL-producing Gram-negative bacterial pathogens. However, their considerable cost and limited availability hinder their use. Also, the combination of ceftazidime-avibactam and aztreonam has been occasionally used, especially when there was an unavailability of aztreonam-avibactam. Additionally, old antibiotics such as colistin and fosfomycin, especially in combination antimicrobial therapy, may be considered for the treatment of patients with such infections. The relevant clinical data on the use of tigecycline and aminoglycosides for infections caused by MBL-producing pathogens are limited. Thus, the evaluation of the published evidence suggests the best treatment options for the treatment of patients with such infections at present. Also, the limited available data highlight the need for dissemination of the already acquired clinical experience and the conduct of new studies examining currently available antibiotics, as well as investigational antimicrobial agents for the treatment of patients with infections caused by MBL-producing Gram-negative bacterial pathogens.

## 1. Introduction

Infections caused by metallo-β-lactamase (MBL)-producing Gram-negative bacteria have become a global public health problem [1]. Additionally, these bacteria are frequently resistant to various antibiotics, resulting in limited therapeutic options. Subsequently, the morbidity and mortality of patients with MBL-producing Gram-negative bacterial infections are significant.

Several traditional antibiotics and non-traditional antimicrobial agents are in the clinical stages of development as therapeutic options for patients with MBL-producing Gram-negative bacterial infections, including those caused by lactose-non-fermenting Gram-negative bacteria, such as *Pseudomonas aeruginosa* and *Acinetobacter baumannii* [2]. However, there are only a few antimicrobial agents currently available in clinical practice that can be used for patients with MBL-producing Gram-negative bacterial infections. Here, we evaluate the antibiotics that could be a valuable option for treating patients with such infections, based on relevant clinical evidence (Table 1).

## 2. Cefiderocol

Cefiderocol is a siderophore cephalosporin. The drug mimics natural siderophore molecules and, through this “Trojan horse strategy,” it enters the periplasmic space via iron transporters, such as the CirA and Fiu for *Escherichia coli* and the PiuA for *Pseudomonas aeruginosa* [3,4]. There, the drug binds to penicillin-binding proteins (PBPs), mainly the PBP3, and inhibits the synthesis of peptidoglycan [3,4].

In a Phase 3 clinical trial (CREDIBLE-CR), 16 patients received cefiderocol and seven received the best-available therapy (six with colistin-based regimens and one with amikacin plus doripenem) for the treatment of infections caused by MBL-producing pathogens [5,6]. Patients who received cefiderocol-based treatment had a numerically higher clinical cure compared to those who received the best-available therapy [12/16 (75%) vs. 2/7 (28.6%)], as well as microbiological eradication [10/16 (62.5%) vs. 1/7 (14.3%)] [5]. 28-day all-cause mortality was numerically lower in the cefiderocol group compared to the other group [1/16 (6.3%) vs. 4/7 (57.1%)] [5].

In a multicenter retrospective study in the US, 10 patients with an infection caused by MBL-producing pathogens [nine New Delhi metallo-β-lactamase (NDM) and one Verona integron-encoded metallo-β-lactamase (VIM)] were treated with cefiderocol [7]. Specifically, 73 patients received monotherapy and 39 patients received combination therapy with cefiderocol (8 with colistin or polymyxin, 7 with ceftazidime-avibactam, 6 with an aminoglycoside, 5 with eravacycline, 5 with imipenem plus cilastatin plus relebactam, 4 with cefepime, and 4 with ampicillin/sulbactam) [7].

In total, 8/10 (80%) patients had a clinical cure; no statistical difference was observed between the monotherapy and combination groups [48/73 (65.8%) vs. 21/39 (53.8%); *p* = 0.24, respectively] [7]. Additionally, none of the patients died within 30 days following treatment [7]. Six out of ten developed on-treatment cefiderocol resistance; four had an infection caused by *Pseudomonas aeruginosa*, one by *Acinetobacter baumannii*, and one by *Klebsiella pneumoniae* [7]. Additionally, two out of ten patients had a 30-day microbiological recurrence [7]. Concerning adverse events, two out of seventeen patients (including those without MBL infections) with a history of allergic reaction to penicillin (ranging from itching with or without hives to angioedema and anaphylaxis) developed a non-anaphylactic rash [7].

In a single-center retrospective study in Spain, 34 patients with infections caused by VIM-producing Gram-negative isolates were treated with cefiderocol [8]. The majority of the patients had respiratory tract infections [9/34 (27%)] and skin and soft tissue infections (SSTIs) [9/34 (27%)], but also urinary tract infections (UTIs) [7/34 (21%)], bone infections [4/34 (21%)], bloodstream infections [3/34 (9%)], and intra-abdominal infections (IAIs) [2/34 (6%)] [8]. In total, 5/34 (14.7%) had clinical failure after 14 days; 30-day mortality was 9/34 (26.5%) [8]. After 3 months, among the 25 patients who survived, 5/25 (20%) had a microbiological recurrence [8].

Additionally, in a prospective study in Italy, 33 patients with infections caused by MBL-producing pathogens received cefiderocol-based regimens [9]. In total, 13 patients received cefiderocol plus fosfomycin, 13 received cefiderocol plus tigecycline, four received cefiderocol monotherapy, and one received cefiderocol plus gentamicin [9]. In this study, the 30-day mortality was 11/33 (33.3%) [9]. Additionally, 3/33 (9.1%) of patients had an adverse event; specifically, one had thrombocytopenia, one had an allergic reaction (not specified), and one had a *Clostridioides difficile* infection [9].

Two studies were presented as conference abstracts and provide relevant data [10,11]. In a study of 24 patients, 16 received cefiderocol and eight received ceftazidime-avibactam plus aztreonam [10]. Cefiderocol treatment showed similar all-cause mortality compared to ceftazidime-avibactam plus aztreonam [3/16 (19%) vs. 2/8 (25%); *p* = 0.72], numerically lower microbiological cure [7/16 (44%) vs. 7/8 (88%); *p* = 0.11], and a trend for shorter length of stay (6.5 ± 3 days vs. 9.5 ± 4 days; *p* = 0.09) [10]. In a retrospective multicenter study, 21 patients received cefiderocol, and 20 patients received ceftazidime-avibactam plus aztreonam for infections caused by MBL-producing pathogens, mainly NDM [15/21 (71.4%) and 16/20 (80%), respectively] [11]. The remaining patients had infections caused by VIM-producing pathogens [11]. Patients who received cefiderocol had a numerically lower clinical cure compared to those who received ceftazidime-avibactam plus aztreonam (71.4% vs. 85%) and a numerically higher 28-day mortality (23.8% vs. 10%; *p* = 0.50) [11].

In a case series of three patients (one of whom had a reinfection) who received cefiderocol for the treatment of UTIs, ventilator-associated pneumonia (VAP), and skin and soft tissue infections (SSTIs) caused by VIM-2-producing *Pseudomonas* species, specifically three *Pseudomonas aeruginosa* and one *Pseudomonas putida* [12]. In this study, all patients achieved a clinical cure [12]. However, one patient experienced persistent colonization with *Pseudomonas aeruginosa* [12].

A patient with an infection following a tibial fracture caused by NDM-producing *Pseudomonas aeruginosa*, with a cefiderocol minimum inhibitory concentration (MIC) of 0.5 mg/L, was treated with cefiderocol for 169 days [for 63 days, the patient received outpatient antimicrobial therapy via a peripheral-inserted central catheter (PICC) line] [13]. Ten days after treatment with cefiderocol, the patient developed iron-deficiency anemia [13]. No acquired resistance to cefiderocol was reported [13]. In another case report, a patient with IAI received cefiderocol for 8 days, showing clinical improvement and a reduction in the inflammatory biomarker C-reactive protein (CRP) (from 201 mg/L to 146 mg/L) [14].

## 3. Aztreonam-Avibactam

Aztreonam is a β-lactam that, by binding to PBPs (specifically PBP3), leads to the disruption of the bacterial membrane and cell death [15]. It is active against bacteria that produce MBLs [15]. On the other hand, avibactam is a β-lactamase inhibitor (not a β-lactam) that inhibits the class A, C, and D β-lactamases, but not the MBLs [15]. The combination of aztreonam-avibactam is potentially effective against all β-lactamases [15].

A Phase 3 clinical trial, the “ASSEMBLE” study (NCT03580044), evaluated the activity of aztreonam-avibactam against Gram-negative pathogens, including those that produce MBLs, compared to best available therapy (BAT) [16]. The trial was conducted from 2020 to 2023 [16]. Patients with complicated intra-abdominal infection (cIAI), including hospital-acquired pneumonia (HAP), VAP, complicated urinary tract infection (cUTI), or bloodstream infection, were included [16]. Patients received intravenous aztreonam-avibactam over a 3 h infusion [16]. Those with cIAI also received 50 mg of intravenous metronidazole every 8 h on a one-hour infusion [16].

In the intent-to-treat (ITT) analysis, there were 12 and 3 participants in the aztreonam-avibactam and the BAT groups, respectively [16]. The mean [±standard deviation (SD)] age was 56.6 (±17.14) for the aztreonam-avibactam group and 65.7 (±6.66) for the BAT group [16]. The patients received therapy for 14 days [16].

The study showed that 5/12 (41.7%) of patients in the aztreonam-avibactam group achieved a clinical cure at the test-of-care visit (28 days after treatment initiation), compared to 0/3 (0%) in the BAT group [16]. At the end-of-treatment (up to 24 h after the last infusion), 7/12 (58.3%) of patients who received aztreonam-avibactam achieved a clinical cure at the test-of-care visit (28 days after treatment initiation), compared to 0/3 (0%) who received the best-available therapy [16]. Within 28 days after treatment initiation, 2/12 (8.3%) died in the aztreonam-avibactam group compared to 1/3 (33.3%) in the BAT group [16]. However, all-cause mortality was 2/12 (16.7%) compared to 1/3 (33.3%), respectively [16]. Serious adverse events were reported in 5/12 (41.7%) patients in the aztreonam-avibactam group and 2/2 (100%) in the BAT group [16].

## 4. Ceftazidime-Avibactam with Aztreonam

The combination of aztreonam-avibactam was approved in April 2024 in the European Union, in June 2024 in the United Kingdom, and in February 2025 in the United States [17,18,19]. However, the synergistic activity of aztreonam and avibactam was known before its release. Thus, ceftazidime-avibactam (an already approved drug) was administered concurrently with aztreonam to achieve the therapeutic effect of the combination of aztreonam with avibactam for classes A, B, C, and D β-lactamases.

In an observational prospective study, 102 patients had infections caused by MBL-producing Enterobacterales (82 NDM and 20 VIM) [20]. In total, 52 patients received ceftazidime-avibactam plus aztreonam, and 50 received other antibiotics [20]. Specifically, 27 received colistin-based regimens (seven colistin plus fosfomycin plus tigecycline, seven colistin plus fosfomycin, five colistin plus meropenem, four colistin plus aztreonam ± piperacillin-tazobactam, two colistin monotherapy, one colistin plus gentamicin, and one colistin plus cotrimoxazole) and 23 received other regimens, not containing colistin (eight tigecycline plus aminoglycosides, five fosfomycin plus aminoglycosides, four aztreonam plus aminoglycosides, two tigecycline plus fosfomycin, two aztreonam monotherapy, one tigecycline plus meropenem, and one aztreonam plus fosfomycin) [20]. In total, patients who received ceftazidime-avibactam plus aztreonam had significantly lower mortality compared to patients who received other antibiotics [10/52 (19.2%) vs. 22/50 (44%); *p* = 0.007] [20].

In another prospective study, 215 patients with infections caused by MBL-producing Enterobacterales pathogens received ceftazidime-avibactam plus aztreonam-based regimens (170 received ceftazidime-avibactam plus aztreonam only, 23 received ceftazidime-avibactam plus fosfomycin, 21 received ceftazidime-avibactam plus tigecycline, and one received ceftazidime-avibactam plus gentamicin) [9]. The 30-day mortality in this study was 48/215 (22.3%) [9].

In a retrospective study, 33 patients had infections caused by NDM plus oxacillinase-48 (OXA-48)-producing *Klebsiella pneumoniae*, and seven by NDM-producing *Escherichia coli* [21]. Among the total of 40 patients, 21 received ceftazidime-avibactam plus aztreonam plus polymyxin, 12 received ceftazidime-avibactam plus aztreonam, and seven received ceftazidime-avibactam plus aztreonam plus fosfomycin [21]. In total, 14/21 (66.7%), 11/12 (91.7%), and 6/7 (85.7%) of patients receiving ceftazidime-avibactam plus aztreonam plus polymyxin, ceftazidime-avibactam plus aztreonam, and ceftazidime-avibactam plus aztreonam plus fosfomycin, respectively, achieved a clinical cure [21].

In a case series, four patients with infections caused by imipenemase-4 (IMP-4)-producing *Enterobacter cloacae* were treated with ceftazidime-avibactam plus aztreonam [22]. Three patients experienced microbiological eradication, and one had a recurrence [22]. In a 40-year-old patient with infection (with an unspecified source) caused by pandrug-resistant *Klebsiella pneumoniae* harboring the *bla*_NDM-1_, *bla*_OXA-48_, *bla*_CTX-M-14b_, *bla*_SHV-28_, and *bla*_OXA-1_ genes, the combination of ceftazidime-avibactam plus aztreonam led to symptom resolution and microbiological eradication [23].

Also, two cases with septic shock caused by NDM-1-producing *Klebsiella pneumoniae* isolates were treated with ceftazidime-avibactam plus aztreonam and had clinical cure [24]. Both cases had recurrence of infection (one month and three months after the end of treatment, respectively), and both were successfully treated with the administration of a second regimen of this drug combination [24]. A pediatric patient with an NDM-1-producing *Morganella morganii* infection received ceftazidime-avibactam (100 mg/kg/day) plus aztreonam (150 mg/kg/day), resulting in recovery and no recurrence after 6 months [25].

In another case report, a patient had sepsis [with a sequential organ failure assessment (SOFA) score of 9] caused by NDM-producing *Klebsiella pneumoniae* [26]. The pathogen was extensively drug-resistant (XDR), as it was susceptible only to colistin (MIC ≤ 0.5 mg/L) [26]. However, the patient had renal failure and did not receive colistin, but received ceftazidime-avibactam (1.5 g) plus aztreonam (1 g) every 8 h, and had clinical and microbiological cure after 10 days [26]. Additionally, a patient with pyelonephritis caused by an NDM-positive, pandrug-resistant (PDR) *Klebsiella pneumoniae* received rescue therapy with cefepime-avibactam plus aztreonam, with recovery after 14 days of treatment [27]. Improvement was reported from the first day of therapy [27]. Another patient with NDM-1-producing *Enterobacter cloacae* infection after a renal transplantation was treated with cefepime-avibactam plus aztreonam [28]. The patient also received other antibiotics, such as posaconazole for *Rhizopus oryzae* complex, and metronidazole for potential infection by anaerobes [28]. The patient had a microbiological eradication after treatment [28].

Additionally, a patient with an infection caused by MBL-producing *Pseudomonas aeruginosa* harboring the *bla*_VIM_ gene with sequence type 235 (ST 235) received ceftazidime-avibactam plus aztreonam and amikacin, achieving microbiological eradication with no recurrence after 12 months [29]. Another patient with an open fracture and an infection caused by VIM-producing *Klebsiella pneumoniae* was treated with ceftazidime-avibactam plus aztreonam for 25 days [30]. The inflammatory marker CRP declined, and the patient had no recurrence after 60 days [30].

## 5. Colistin

Colistin is a polymyxin that is cationic and binds to the negatively charged lipid A portion of the lipopolysaccharides (LPS) of the outer bacterial membrane [31,32]. The Ca^2+^ and Mg^2+^ ions are displaced, resulting in an increase in permeability of the bacterial membrane and, ultimately, bacterial death [31,32].

In a retrospective study, seven patients with an infection caused by MBL-producing *Klebsiella pneumoniae* (four with IMP and three with NDM) received colistin plus ceftazidime-avibactam [33]. The 30-day mortality was 2 out of 7 patients, one with IMP and one with an NDM-producing pathogen [33]. All survivors had a respiratory tract infection, and 3 out of 5 had a microbiological cure in seven days [33].

In a prospective observational study, 26 patients with infections caused by MBL-producing Enterobacterales received colistin-based regimens (14 patients colistin plus tigecycline ± fosfomycin, six colistin plus fosfomycin, four colistin monotherapy, one colistin plus piperacillin-tazobactam plus aztreonam, and one colistin plus aztreonam) [9]. The 30-day mortality in this study was 13/26 (50%) [9]. Concerning adverse events, 9/26 (34.6%) of patients receiving colistin had acute kidney injury [9]. The proportion of adverse events was higher in the colistin-based group compared to other combinations (*p* < 0.001) [9].

In another prospective observational study, 27 patients received colistin-based regimens [20]. Specifically, seven patients received colistin plus fosfomycin plus tigecycline, seven colistin plus fosfomycin, five colistin plus meropenem, four colistin plus aztreonam ± piperacillin-tazobactam, one colistin plus gentamicin, one colistin plus cotrimoxazole, and one colistin monotherapy [20]. The 30-day mortality in this study was 16/27 (59.3%) [20].

## 6. Fosfomycin

Fosfomycin is a phosphonic acid derivative [34]. It inhibits the formation of UDP *N*-acetylmuramic acid by binding to the active site of the enzyme UDP-*N*-acetylglucosamine enolpyruvyl transferase (MurA) [34]. Thus, fosfomycin interferes with the first step in bacterial cell wall synthesis [34].

In a prospective observational study, 22 patients received therapy for infections caused by MBL-producing Enterobacterales with fosfomycin-containing regimens (15 received fosfomycin monotherapy, five received fosfomycin plus tigecycline, one received fosfomycin plus gentamicin, and one received fosfomycin plus meropenem) [9,35]. In total, 30-day mortality was 4/22 (18.2%), specifically three patients who received fosfomycin monotherapy and one who received fosfomycin plus tigecycline [9,35].

In another observational prospective study, patients received various combinations that included fosfomycin for the treatment of bloodstream infections caused by MBL-producing Enterobacterales [20]. The 30-day mortality was 6/7 (85.7%) for those who received colistin plus fosfomycin plus tigecycline, 5/7 (71.4%) for those who received colistin plus fosfomycin, 0/5 for those who received fosfomycin plus aminoglycosides, and 2/2 for those who received fosfomycin plus tigecycline [20].

## 7. Tigecycline

Tigecycline is a glycylycline-class antibiotic (a tetracycline derivative) [36]. It acts by binding to the ribosomal 30S subunit, thus inhibiting protein synthesis [36]. In a retrospective study in Spain, 15 patients were treated for infections caused by VIM-1-producing *Klebsiella pneumoniae* [37]. There were a total of 16 infectious episodes [37]. All isolates were susceptible to tigecycline, with an MIC of <2 mg/L [37]. In total, 10 episodes were treated with 100 mg of tigecycline administered every 12 h, and six episodes were treated with 50 mg of tigecycline administered every 12 h [37]. Six episodes were treated with tigecycline plus colistin, two with tigecycline monotherapy, two with tigecycline plus imipenem, one with tigecycline plus colistin plus meropenem, one with tigecycline plus colistin plus amikacin, one with tigecycline plus meropenem, one with tigecycline plus meropenem plus ciprofloxacin, one with tigecycline plus ciprofloxacin, and one with tigecycline plus piperacillin-tazobactam [37]. Microbiological eradication was achieved in 5/16 (31.3%) of the infection episodes [37]. The 30-day mortality in this study was 4/16 (25%) [37].

In a small case series, two patients received tigecycline in combination with colistin for infections caused by MBL-producing bacteria [38]. One patient had urinary tract and bloodstream infections caused by an XDR *Klebsiella pneumoniae* pathogen that produced the cefotaxime-M β-lactamase (CTX-M), OXA-48, and NDM β-lactamases [38]. The patient received high-dose tigecycline (200 mg loading dose followed by 100 mg every 12 h) and colistin for 14 days [38]. During treatment, the patient had nausea, vomiting, and diffuse abdominal pain; however, the symptoms resolved with supportive care [38]. At the end of therapy, the patient had a clinical and microbiological cure [38].

Another patient had a UTI caused by a PDR *Klebsiella pneumoniae* that was producing extended-spectrum-β-lactamase (ESBL) and MBL β-lactamases [38]. The patient received high-dose tigecycline (200 mg loading dose followed by 100 mg every 12 h) and colistin [nine million international units (mIU) loading dose, then three mIU every eight hours] for seven days [38]. During therapy, the patient had nausea, dizziness, loss of appetite, and diffuse abdominal pain; however, the symptoms resolved with symptomatic treatment [38]. The patient had a clinical and microbiological cure [38].

In a case report, a patient was treated for a bone/soft tissue infection and bacteremia caused by a *bla*_NDM-5_-producing *Escherichia coli*, with tigecycline plus colistin for 10 days, followed by tigecycline plus cefiderocol for a total of four weeks [39]. The patient had microbiological eradication during hospitalization but had a recurrence about two months later [39].

## 8. Aminoglycosides

Aminoglycosides, such as amikacin, gentamicin, tobramycin, and plazomicin, inhibit bacterial protein synthesis by binding to the ribosomal 30S subunit [40]. In a case report, a patient with bloodstream infection and UTI caused by an NDM-producing *Klebsiella pneumoniae* [41]. The patient received plazomicin, a new synthetic aminoglycoside, combined with meropenem-vaborbactam for seven days [41]. Then, the patient was transferred to another facility and received ceftazidime-avibactam with tigecycline for one day, followed by omadacycline, a new tetracycline, specifically an aminomethylcycline, plus tigecycline for another day [41]. Next, as the drug combination of plazomicin combined with meropenem-vaborbactam was available, the patient received these drugs for 14 days [41]. Microbiological eradication was achieved, and there was no recurrence 6 months after treatment [41].

## 9. Discussion

The evaluation of the published evidence shows that there is limited clinical information on the effectiveness and safety of various antimicrobial agents for the treatment of patients with infections caused by MBL-producing pathogens. The available data suggest that clinicians may consider new antibiotics, specifically cefiderocol and aztreonam-avibactam, but also old antibiotics, specifically colistin, for the treatment of patients with such infections.

The new antibiotics may be preferred compared to colistin, given the nephrotoxicity of polymyxins and the limited effectiveness in patients with pulmonary infections. However, cost and limited availability are essential obstacles to the use of these new antibiotics. There is also some supporting clinical data on the combination of ceftazidime-avibactam and aztreonam for patients with infections caused by MBL-producing Gram-negative bacterial pathogens. However, this combination has been used by clinicians where there was no availability of aztreonam-avibactam. The combination of ceftazidime-avibactam and aztreonam does not offer a clear advantage over the use of aztreonam-avibactam, given that there are no data to support a synergistic effect between ceftazidime and aztreonam-avibactam.

Additionally, although clinical data on the use of fosfomycin for the treatment of patients with infections caused by MBL-producing pathogens are limited, the available studies demonstrate that fosfomycin could be valuable, especially in combination with antimicrobial therapy, in cases where other relevant treatment options (such as aztreonam-avibactam) are not available. Fosfomycin has exhibited considerable synergistic activity in in vitro studies with several antimicrobial agents [42,43]. However, outcomes such as clinical cure have different definitions in each study, resulting in increased heterogeneity among studies that report this outcome.

The data on the use of tigecycline for the treatment of patients with infections caused by MBL-producing Gram-negative bacterial pathogens are limited. They suggest considering this antibiotic based on supporting in vitro antimicrobial susceptibility testing results, within the approved indications of the antibiotic, and usually in combination with antimicrobial treatment.

Regarding aminoglycosides, there is very limited published data on the use of this class of antimicrobial agents for the treatment of patients with infections caused by MBL-producing pathogens. Subsequently, there is no scientific ground at this time to suggest aminoglycosides for the treatment of patients with such infections, given also their considerable nephrotoxicity.

A scenario of particular concern in clinical practice is the administration of a potentially active antimicrobial agent for the treatment of a patient with an infection caused by an MBL-producing Gram-negative bacterial pathogen that has been proven to be resistant based on in vitro antimicrobial susceptibility testing. Additionally, another concern is the development of resistance of the pathogen to the administered agent, although it was initially susceptible to the treatment. These concerns are pertinent for all antimicrobial agents that are considered for the treatment of patients with infections caused by MBL-producing Gram-negative bacterial pathogens, including cefiderocol, aztreonam-avibactam, colistin, fosfomycin, tigecycline, and aminoglycosides [42,44,45]. These concerns also underscore the importance of ongoing, meticulous evaluation of the patient, including the performance of in vitro susceptibility tests on samples from the affected infection sites. 

A limitation of the available literature on the treatment options for patients with infections caused by MBL-producing Gram-negative bacterial pathogens is that the majority of the relevant studies were observational (retrospective or prospective), as well as case series and case reports. Only one clinical trial was available for inclusion in our article that focused on aztreonam-avibactam. These observational studies reflect the use of the various antimicrobial agents in real-world, pragmatic conditions, a condition not usually fulfilled in randomized controlled trials [46]. Still, due to the lack of randomization, they may have a higher risk of bias, especially confounding, selection, and publication bias. Additionally, the case series as well as case reports have inherent limitations, such as the inability to adjust for confounding factors, lack of a comparison group, and a potentially high risk of selection and publication bias. On contrast, randomized controlled trials have higher internal validity due to their study characteristics, specifically randomization, the presence of a control group, and single or double blinding (of participants and/or researchers).

## 10. Conclusions

There are limited published data for the management of patients with infections caused by MBL-producing Gram-negative bacterial pathogens. The new antibiotics cefiderocol and aztreonam-avibactam, as well as the old antibiotics colistin and fosfomycin, are potential therapeutic options to consider. The scarcity of published data on this emerging, significant public health problem necessitates the dissemination of available data with publication of relevant experience and the conduct of studies of potential options for the treatment of patients with infections caused by MBL-producing Gram-negative bacterial pathogens.

## Figures and Tables

**Table 1:** Therapeutic options for the treatment of patients with infections caused by MBL-producing Gram-negative bacterial pathogens.

**Limited Clinical Data**
*New antibiotics*
Cefiderocol
Aztreonam-avibactam
Ceftazidime-avibactam and aztreonam (if aztreonam-avibactam is unavailable)
*Old antibiotics*
Colistin
Fosfomycin
**Very limited clinical data**
*Old antibiotics*
Tigecycline
Aminoglycosides

## Abbreviations

BAT	best available therapy
cUTI	complicated urinary tract infection
CRP	C-reactive protein
CTX-M	cefotaxime-M β-lactamase
ESBL	extended-spectrum-β-lactamase
HAP	hospital-acquired pneumonia
IAI	intra-abdominal infection
IMP-4	imipenemase-4
ITT	intent-to-treat
LPS	lipopolysaccharides
MBL	metallo-β-lactamase
MIC	minimum inhibitory concentration
mIU	million international units
NDM	New Delhi metallo-β-lactamase
OXA-48	oxacillinase-48
PBP	penicillin-binding protein
PDR	pandrug-resistant
PICC	peripheral-inserted central catheter
SOFA	sequential organ failure assessment
SSTI	skin and soft tissue infection
ST 235	sequence type 235
UTI	urinary tract infection
VAP	ventilator-associated pneumonia
VIM	Verona integron-encoded metallo-β-lactamase
XDR	extensively drug-resistant
